# Assessment of Healthcare Professionals' Self-Perceived Competence in Perinatal/Neonatal Palliative Care After a 3-Day Training Course

**DOI:** 10.3389/fped.2020.571335

**Published:** 2020-09-22

**Authors:** Jennifer Hammond, Charlotte Wool, Elvira Parravicini

**Affiliations:** ^1^Division of Neonatology, Department of Pediatrics, Columbia University Irving Medical Center, New York, NY, United States; ^2^Department of Nursing, York College of Pennsylvania, York, PA, United States

**Keywords:** perinatal, neonatal, palliative care, competence, training course

## Abstract

**Background:** Perinatal/neonatal palliative care (PNPC) offers a plan of care for improving the quality of life of infants when the prolongation of life is no longer the goal of care. The number of PNPC programs has increased in recent years, but training for clinicians has not kept pace. Therefore, an interdisciplinary team developed a 3-day intensive PNPC training course for physicians, nurses, and other healthcare professionals at Columbia University Irving Medical Center (CUIMC).

**Objective:** The aim of this study was to assess the efficacy of a PNPC training course in improving the self-reported competence of participants.

**Study Design:** A cross-sectional survey design was used to obtain data from 88 healthcare professionals who attended the PNPC training course. Data was collected using a validated questionnaire. The questionnaire included 32 items that queried participants about their self-assessed competence using a forced 1–4 Likert scale. The 32 items, which served as the outcome variables, were clustered into the eight domains of palliative care. The survey was administered through a web-based tool at the beginning and the conclusion of the course.

**Results:** Results from two-sample *t*-tests comparing pre-test and post-test self-assessed competence were statistically significant for each item across disciplines. Additional analysis revealed that after participation in the training course, the statistically significant differences between physicians' and nurses' pre-course self-reported competence disappeared.

**Conclusion:** The development of an evidence-based curriculum improved the self-reported competence of participants across disciplines, filled a specific gap in nurses' self-reported competence and addressed a global training need.

## Introduction

Perinatal/neonatal palliative care (PNPC) offers a plan of care for improving the quality of life of infants when the prolongation of life is no longer the goal of care or the complexity of the medical condition is associated with an uncertain prognosis. Palliative care in the perinatal period is a coordinated, interdisciplinary approach to obstetric and newborn care that includes a focus on maximizing the quality of life and comfort of newborns with life-limiting conditions (LLC) (1). This focus extends to supportive, coordinated care for parents and family members by providing anticipatory guidance, honoring parents' values, providing targeted consultations, formulating birth and treatment plans for the infant, and offering bereavement counseling.

The number of perinatal palliative care programs has increased in recent years (2, 3), but training and education for clinicians has not kept pace. There remains a gap in knowledge and competence regarding the management of parents expecting an infant with a LLC during pregnancy and birth and after delivery (4, 5). Furthermore, healthcare professionals from different backgrounds and disciplines have varying experiences, training, and confidence with regards to the delivery of PNPC (6–8). While suggestions for education and training have been proposed (9), formal professional coursework for PNPC is still very limited (10).

A policy statement from the American Academy of Pediatrics (11) on pediatric palliative and hospice care recommends that palliative care should be a part of all pediatric education, training curricula, and quality improvement. In addition, the National Consensus Project (NCP) Clinical Practice Guidelines for Quality Palliative Care states that all clinicians in direct care of seriously ill patients should have the necessary training and experience to complete palliative assessments and address common sources of suffering (12).

In response to clinicians' educational needs and recommendations from national organizations, an interdisciplinary team developed a 3-day intensive training course at Columbia University Irving Medical Center (CUIMC).

This study had two specific aims. First, the study aimed to evaluate the efficacy of the PNPC training course in improving the self-reported competence of course participants in perinatal and neonatal palliative care. Second, the study aimed to examine differences between physicians' and nurses' self-reported competence.

## Materials and Methods

### Training Course

A 3-day course entitled *Next-Level Perinatal/Neonatal Comfort Care Training Course: Developing a Medical and Interdisciplinary Plan for Each Baby and Support for Their Family* was offered at CUIMC in June 2019. The program was designed by a team of medical experts to cover all aspects of PNPC (13). The target audience included the entire perinatal team: physicians, nurses, nurse practitioners, and other allied health professionals practicing in the perinatal arena (i.e., obstetrics, neonatology) who were interested in improving the practice of PNPC both nationally and internationally. Participants were provided with educational resources including a syllabus with objectives and literature references for each session. The course included presentations from an interdisciplinary team of clinicians including physicians, nurses, and researchers from neonatology and obstetrics as well as other professionals (a social worker, psychologist, speech-language pathologist, child life professional, and chaplain) involved in perinatal care and parent interviews. Faculty presented evidence-based rationale and strategies, offered interactive learning opportunities, modeled elements of effective communication, and practiced hands-on activities with participants. These educational strategies were administered to demonstrate and teach family-centric support and the attainment of a state of comfort for newborns with LLC. The eight domains of palliative care published by the NCP (12) were woven into the course curriculum, which included the following topics:

(1) The current state of the science on perinatal palliative care
(2) Strategies to assist families during decision-making processes
(3) Definition and roles of the members of the interdisciplinary team
(4) Essential elements of the perinatal palliative consult
(5) Advance care planning
(6) Evidence-based interventions in the prenatal, intrapartum, and postnatal periods that promote the health of the mother and family
(7) Essential elements of a neonatal palliative care plan
(8) Identification of potential barriers to institutional uptake of perinatal palliative care.

### Study Survey

A survey was developed to assess participants' self-reported competence before and after the course and included 50 items with three sections. Section one included demographic data. Section two contained the outcome variables, which were 32 items addressing self-reported competence (Table 1). Participants were asked to rate their competence on a forced Likert scale of one (not competent), two (somewhat competent), three (competent), and four (highly competent). Participants could select “not applicable” if they did not feel as though they could assess their competence for a specific item. Competence was defined as the “habitual and judicious use of communication, knowledge, technical skills, clinical reasoning, emotions, values and reflection in daily practice for the benefits of the individual and the community being served” (14). The 32 items addressing competence were derived using a two-pronged approach. First, the literature was reviewed and a validated questionnaire by Stekenes and colleagues (15) was modified with permission to create the 32 outcome variables in the survey. The items were examined by two neonatologists and a researcher, reworded where needed, and agreement was reached regarding the inclusion of the final items. Next, the NCP's eight domains of quality palliative care were used to organize items and ensure that each domain was represented with at least one item. Section three queried participants' previous education in palliative care.

**Table 1:** Items assessing self-reported competence grouped by palliative care domain (12).

**Domain**	**Survey Items**
Domain 1: Structure and Process	1. Offering the parent(s) the option of palliative care when they are making decisions about a fetus with a life-limiting condition 2. Talking to parent(s) about the range of options including palliative care after their fetus/infant has been diagnosed with a serious medical condition and potential adverse outcome 3. Talking to parent(s) about the range of possible outcomes after their fetus/infant has been diagnosed with a life-limiting condition 4. Engaging in shared decision-making after a life-limiting diagnosis is confirmed 5. Including parent(s) to create a plan of care for the pregnancy and birth 6. Including parent(s) to create a plan of care for the infant after birth 7. Providing prenatal care in a consistent way 8. Communicating with families about priorities of care 9. Understanding the role of palliative care and its integration into intensive care in the NICU 10. Accessing palliative care expertise and support when needed
Domain 2: Physical	11. Attending the delivery and providing palliative care to an infant with a life-limiting condition 12. Assessing the basic needs of infants with life-limiting conditions 13. Making a comprehensive care plan to address an infant's basic needs 14. Developing a nutrition plan for an infant with a life-limiting condition who is breathing and stable on room air 15. Providing interventions to achieve an infant's comfort 16. Assessing pain/discomfort in infants 17. Managing pain/discomfort in infants 18. Assessing other symptoms in infants (respiratory distress, irritability) 19. Managing other symptoms in infants (respiratory, irritability) 20. Following through with documentation of goals of care for an infant with a serious condition and potential adverse outcomes
Domain 3: Psychological and Psychiatric	21. Addressing the unborn child by name 22. Giving parent(s) a sense of normalcy during pregnancy and birth 23. Providing emotional support to parent(s) expecting an infant with a life-limiting condition 24. Exploring with families how they talk with their other children about the infant who is not expected to live
Domain 4: Social	25. Creating a comfortable, caring environment for parent(s) 26. Accessing resources related to perinatal palliative care (clinical resource materials, continuing education) 27. Knowing the community resources available for palliative care and how to access these resources
Domain 5: Religious, Spiritual, Existential	28. Sensitivity to spiritual needs
Domain 6: Cultural	29. Sensitivity to cultural values
Domain 7: Care of the Imminently Dying	30. Having conversations with families about the possibility of their infant dying 31. Recognizing signs of impending death in an infant
Domain 8: Legal and Ethical	32. Identifying and understanding ethical issues related to end of life care for infants

### Study Design

The study was approved by the Institutional Review Board at CUIMC (AAAS4060) and informed consent was obtained from participants at the start of data collection. In order to assess the course outcomes, a pre-test, post-test study was developed. The pre-test, post-test design was cross-sectional and data were collected at two discrete points in time: before the training course commenced and after the course was completed. At the start of the course, a link to the pre-test survey was sent to each participant's electronic inbox using the web-based tool Qualtrics. An introduction to the survey and informed consent were provided on the first page. The training course was considered the intervention and lasted a total of 3 days. At the close of the training course, a link to the survey was emailed to participants. Follow-up emails to solicit completion of the post-test survey were sent 1 and 3 weeks after the conclusion of the course.

### Statistical Analysis

Prior to performing the analyses, “not applicable” responses for each item were removed from the dataset and therefore not included in the final analyses. In order to examine differences between pre-test and post-test self-reported competence, each of the thirty-two items were compared using a two-sample *t*-test assuming unequal variance. Next, the items were clustered into their respective palliative care domains. The means for each item in each clustered domain were compared using a two-sample *t*-test assuming unequal variance. In addition, an independent sample *t*-test was completed to examine the difference in the self-reported competence between physicians and nurses at the start of and end of the training course.

## Results

A total of 88 professionals from nineteen US states and six countries (Australia, Burundi, Canada, England, Italy, and Russia) attended the course; however, only 76 attended the complete 3-day course and qualified for participation in the study. The pre-test survey response rate was 100% and the post-test survey response rate was 67%. Most participants were physicians or nurses and mainly represented the fields of neonatology and obstetrics. One third of participants had limited clinical palliative care experience (cared for fewer than five dying infants in their career), while 47% of participants reported having some previous exposure to palliative care education. Demographic data are presented in Table 2.

**Table 2:** Demographic characteristics of participants.

**Demographic Characteristics**	**Number of Participants (%)**
**Profession**	
Nurse	34/76 (45)
Midwife	2/76 (3)
Physician	31/76 (41)
Other	9/76 (11)
**Area of Practice**	
Neonatology	49/76 (65)
Obstetrics	9/76 (12)
Palliative Care	8/76 (10)
Pediatrics	3/76 (4)
Other	7/76 (9)
**Highest Level of Education**	
High School Diploma	2/76 (3)
Undergraduate Degree	24/76 (31)
Master's Degree	21/76 (28)
Doctorate	29/76 (38)
**Age**	
<30 years	13/76 (17)
31–40 years	32/76 (42)
41–50 years	11/76 (14.5)
51–60 years	11/76 (14.5)
61–70 years	9/76 (12)
**Gender**	
Male	16/76 (22)
Female	60/76 (78)
**Country of Origin**	
USA	62/76 (82)
Other	14/76 (18)

Analysis of the 32 items assessing self-reported competency showed that pre-course and post-course self-reported competence scores were statistically different for all professionals for each of the 32 items with higher scores on the post-course survey (Table 3). Comparisons remained statistically significant when the items were clustered into their respective palliative care domains.

**Table 3:** Pre-course and post-course self-assessed competency for all participants.

**Item number**	***N* (Pre-test)**	***N* (Post-test)**	**Pre-test M (sd)**	**Post-test M (sd)**	***t*-test (df); sig**.
1	63	44	2.86 (0.99)	3.50 (0.35)	−4.17 (103); *p* < 0.001
2	63	46	2.95 (1.01)	3.48 (0.34)	−3.43 (103); *p* < 0.001
3	64	43	2.81 (0.95)	3.44 (0.40)	−4.06 (105); *p* < 0.001
4	64	44	3.00 (0.86)	3.55 (0.35)	−3.74 (105); *p* < 0.001
5	57	44	2.49 (1.22)	3.57 (0.48)	−5.99 (96); *p* < 0.001
6	61	43	2.77 (1.15)	3.70 (0.26)	−5.88 (92); *p* < 0.001
7	53	45	2.57 (1.10)	3.40 (0.65)	−4.44 (95); *p* < 0.001
8	68	42	2.96 (0.88)	3.57 (0.40)	−4.11 (107); *p* < 0.001
9	72	42	2.97 (0.90)	3.76 (0.23)	−5.87 (110); *p* < 0.001
10	68	40	2.88 (0.97)	3.63 (0.29)	−5.05 (106); *p* < 0.001
11	64	41	2.95 (0.93)	3.61 (0.44)	−4.11 (102); *p* < 0.001
12	70	41	2.97 (0.98)	3.56 (0.40)	−3.81 (108); *p* < 0.001
13	69	38	2.86 (0.86)	3.61 (0.30)	−5.26 (104); *p* < 0.001
14	66	40	2.38 (1.07)	3.43 (0.51)	−6.16 (102); *p* < 0.001
15	69	39	2.91 (1.08)	3.64 (0.24)	−4.94 (103); *p* < 0.001
16	67	39	3.09 (0.96)	3.56 (0.30)	−3.19 (104); *p* = 0.001
17	66	37	3.05 (0.97)	3.54 (0.26)	−3.37 (100); *p* = 0.001
18	67	42	3.19 (0.92)	3.67 (0.28)	−3.32 (105); *p* = 0.001
19	63	39	3.16 (0.88)	3.59 (0.30)	−2.93 (100); *p* = 0.004
20	70	43	2.77 (0.93)	3.58 (0.30)	−5.70 (110); *p* < 0.001
21	53	38	3.32 (0.99)	3.84 (0.30)	−3.20 (84); *p* = 0.001
22	57	42	2.92 (1.00)	3.60 (0.39)	−4.06 (95); *p* < 0.001
23	56	43	2.91 (0.88)	3.56 (0.44)	−4.01 (96); *p* = 0.001
24	72	46	2.33 (0.87)	3.46 (0.48)	−7.49 (113); *p* < 0.001
25	65	41	3.31 (0.75)	3.73 (0.25)	−3.19 (103); *p* = 0.001
26	71	47	2.69 (0.87)	3.60 (0.33)	−6.50 (116); *p* < 0.001
27	73	48	2.55 (0.81)	3.29 (0.51)	−5.05 (115); *p* < 0.001
28	65	43	3.12 (0.58)	3.67 (0.22)	−4.64 (106); *p* < 0.01
29	67	44	3.09 (0.60)	3.68 (0.22)	−5.01 (108); *p* < 0.01
30	68	43	3.01 (0.91)	3.53 (0.45)	−3.38 (108); *p* < 0.001
31	66	41	3.02 (0.94)	3.63 (0.33)	−4.13 (105); *p* < 0.001
32	72	46	2.96 (0.69)	3.68 (0.22)	−5.01 (108); *p* < 0.01

Self-reported competency was then analyzed by discipline. The post-course self-reported competence scores for nurses were statistically higher than their pre-course scores for each of the 32 items. Physicians' post-course self-reported competence scores were higher than their pre-course scores and reached statistical significance for 20 of the 32 items including items 1 (*p* = 0.03), 5 (*p* = 0.03), 6 (*p* = 0.01), 8 (*p* = 0.02), 9 (*p* < 0.001), 10 (*p* = 0.005), 11 (*p* = 0.04), 12 (*p* = 0.03), 13 (*p* < 0.001), 14 (*p* = 0.001), 15 (*p* < 0.001), 16 (*p* = 0.004), 20 (*p* = 0.004), 24 (*p* < 0.001), 26 (*p* < 0.001), 27 (*p* = 0.008), 28 (*p* = 0.002), 29 (*p* = 0.001), 30 (*p* < 0.001), 32 (*p* = 0.002).

Scores for nurses and physicians were also directly compared. Prior to the start of the course physicians' mean scores for self-reported competence were higher than the nurses' mean scores for nine items. In post-course analyses, the nine self-reported competence items were again compared between nurses and physicians and were no longer statistically significant (Table 4).

**Table 4:** Comparison of physician and nurse differences in self-reported competency in pre-test and post-test items.

	**Nurses**			**Physicians**			
	**Mean**	**SD**	***N***	**Mean**	**SD**	***N***	***t*-test (df); sig**.
**Pre-test Items**							
Offering parents options	2.53	1.00	30	3.07	0.92	29	−2.12 (57), *p* = 0.03
Talking about options	2.61	1.05	31	3.18	0.94	28	−2.16 (57), *p* = 0.03
Talk about outcomes	2.39	0.98	31	3.21	0.77	29	−3.55 (58), *p* = 0.001
Engaging in shared decision-making	2.65	1.01	31	3.28	0.79	29	−2.65 (58), *p* = 0.01
Consistent prenatal care	2.17	1.07	23	2.85	0.96	26	−2.30 (47), *p* = 0.02
Access palliative expertise/support	2.50	0.99	34	3.18	0.90	28	−2.78 (60), *p* = 0.007
Nutrition plan for infant	2.09	1.05	32	2.70	0.95	31	−2.36 (60), *p* = 0.02
Follow through with documentation	2.51	1.01	35	3.00	0.78	30	−2.17 (62), *p* = 0.03
Conversations about dying	2.68	1.03	34	3.28	0.79	29	−2.59 (60), *p* = 0.01
**Post-test Items**							
Offering parents options	3.45	0.67	22	3.53	0.61	19	−0.35 (39), *p* = 0.72
Talking about options	3.45	0.67	22	3.48	0.60	21	−0.11 (41), *p* = 0.91
Talk about outcomes	3.40	0.68	20	3.52	0.60	21	−0.61 (39), *p* = 0.54
Engaging in shared decision-making	3.40	0.68	20	3.67	0.58	21	−1.35 (39), *p* = 0.18
Consistent prenatal care	3.30	0.80	20	3.55	0.80	22	−0.99 (40), *p* = 0.32
Access palliative expertise/support	3.47	0.69	19	3.71	0.47	17	−1.18 (32), *p* = 0.24
Nutrition plan for infant	3.42	0.69	19	3.32	0.82	19	−0.42 (36), *p* = 0.67
Follow through with documentation	3.47	0.69	19	3.65	0.48	20	−0.91 (32), *p* = 0.36
Conversations about dying	3.33	0.84	18	3.71	0.56	21	−1.63 (29), *p* = 0.11

## Discussion

This study found that the development and implementation of an evidence-based PNPC training course improves the self-reported competence of its participants across disciplines and in each domain of palliative care. Moreover, participation in the course filled a specific gap in nurses' self-reported competence.

The improvement in the self-reported competence in each item and in each domain of palliative care demonstrates the breadth of the material presented in the training course. The comprehensive training program addressed not only the medical aspects of perinatal palliative care but also the psychological, spiritual, cultural, and ethical aspects of care. The pre-course mean self-reported competence scores were particularly low for participants across disciplines on several items related to the psychological and social aspects of care. Participants reported the lowest self-reported competency scores in the areas of developing a nutrition plan and communication with the siblings of infants with a life-limiting condition. The creation of a nutrition plan is rarely included in PNPC education because most infants with a life-limiting condition have a short life expectancy. However, some infants may be stable at birth and continue to live for days, weeks, or months. Therefore, hunger and thirst must be addressed (16). Furthermore, colostrum care and non-nutritive sucking are recommended by the American Academy of Pediatrics as non-pharmacological interventions to alleviate pain and discomfort in neonates, including at the end of life (11, 17). Although PNPC emphasizes the care and support of the entire family, training is often lacking in this specific area, which was highlighted by the scores in the “somewhat competent” range for communication with siblings. Participation in this PNPC training course adequately addressed these topics as demonstrated by the marked improvement in the post-course self-reported competence scores.

Additional analyses revealed that nurses improved their self-reported competency scores on each item, while physicians improved their scores in 20 of the 32 items. The domains of palliative care most impacted by course participation were related to the integration of palliative care into NICU practices, addressing infants' basic needs, providing interventions to achieve infants' comfort, accessing resources for PNPC and initiating conversations about dying. Future training programs should continue to incorporate these findings to address the specific educational needs of physicians.

Prior to the start of the course, a statistically significant difference between physicians and nurses self-reported competence was calculated for nine items. These items were in three of the eight domains including the structure and process of care, physical aspects of care, and care of the imminently dying and reflect areas of PNPC that physicians often have more experience with. These findings were not surprising since previous work has suggested that physicians are often more confident than nurses in the practice of perinatal and neonatal palliative care (18, 19). After participation in the training course, these differences disappeared. This suggests that an intensive training course can fill a gap in nurses' self-reported competence in the practice of PNPC.

The training course included participants from different disciplines including neonatology, obstetrics, and palliative care. This is particularly important because PNPC practices require an interdisciplinary team of practitioners. Neonatologists are often at the forefront of neonatal palliative care initiatives and obstetricians' interest in acquiring competence in this field is growing (1). This growth is important since obstetricians are often on the front line of delivering news of a fetal diagnosis of a LLC to parents and providing recommendations.

The implementation of this training course addressed an international need. A recent study found that nearly half of the neonatal intensive care units surveyed did not have neonatal comfort care guidelines and 91% of respondents stated that their institution would benefit from PNPC education (20). Previous work suggests that appropriate PNPC education can increase knowledge, self-perceived confidence, and clinical skills (21). Insufficient knowledge about PNPC is a known barrier to its uptake (6). Furthermore, comprehensive training may alleviate some of the distress, helplessness, and discomfort clinicians have reported in previous studies (8). Finally, numerous nationally recognized organizations including the NCP, ACOG and the Worldwide Palliative Care Alliance have recommendations encouraging clinicians and institutions to develop PNPC programs, which can only be done well when there is ample training for clinicians (1, 12, 22). The CUIMC course attracted individuals from several countries. Importantly, despite the vast and diverse demographic origins of the participants, the course content universally improved professionals' self-reported competence in PNPC management.

The present study has several strengths. First, participants represented several disciplines and specialties which contrasts with previous work. Knighting and colleagues evaluated the impact of a training course on self-perceived knowledge and clinical practice in 73 professionals. However, 82% of participants had a nursing background and only three participants were from the field of obstetrics (21). A second strength of the study includes the timing of the administration of the post-course survey. Since participants completed the post-test survey within 1–3 weeks of course completion, it is unlikely that the improvement in self-reported competence can be explained by additional education or experiences outside of the training course.

There are several limitations of the study. In contrast to the study by Knighting and colleagues, participants' pre-course and post-course responses were not matched and fewer participants completed the post-course survey. In addition, the study focused on measures of self-perceived confidence but did not directly assess knowledge and skills. Finally, since this is a cross-sectional study, it does not measure participants' experiences after return to their healthcare systems. It is unknown if the course materials provided professionals with the ability to develop or improve PNPC programs. Therefore, longitudinal research is recommended to measure outcomes in clinical practice environments.

The development and implementation of an evidence-based PNPC course improved the self-reported competence of participants across disciplines and addressed specific self-reported competence gaps of nurses. This study provides a framework for the establishment of a PNPC curriculum, which addresses a global need.

## Data Availability Statement

The raw data supporting the conclusions of this article will be made available by the authors, without undue reservation.

## Ethics Statement

The studies involving human participants were reviewed and approved by Columbia University Irving Medical Center Institutional Review Board. The patients/participants provided their written informed consent to participate in this study.

## Author Contributions

JH, CW, and EP developed the survey, analyzed the data, and wrote the manuscript. All authors contributed to the article and approved the submitted version.

## Conflict of Interest

The authors declare that the research was conducted in the absence of any commercial or financial relationships that could be construed as a potential conflict of interest.
